# Use of MALDI-TOF MS for Identification of Nontuberculous *Mycobacterium* Species Isolated from Clinical Specimens

**DOI:** 10.1155/2015/854078

**Published:** 2015-05-28

**Authors:** María Concepción Mediavilla-Gradolph, Inmaculada De Toro-Peinado, María Pilar Bermúdez-Ruiz, María de los Ángeles García-Martínez, María Ortega-Torres, Natalia Montiel Quezel-Guerraz, Begoña Palop-Borrás

**Affiliations:** ^1^Infectious Diseases and Microbiology Service, Regional University and Virgen de la Victoria University Hospitals, IBIMA, 29010 Malaga, Spain; ^2^Microbiology Unit, Hospital Costa del Sol, 29603 Marbella, Spain

## Abstract

The aim of this study was to compare the results obtained for identification by MALDI-TOF of nontuberculous mycobacteria (NTM) isolated in clinical samples with those obtained by GenoType *Mycobacterium* CM/AS (common mycobacteria/additional species). A total of 66 *Mycobacterium* isolates from various clinical specimens (mainly respiratory) were tested in this study. They were identified using MALDI-TOF Bruker from strains isolated in Lowenstein, following the recommended protocol of heat inactivation and extraction, and were simultaneously analyzed through hybridization by GenoType *Mycobacterium* from liquid culture MGIT. Our results showed that identification by MALDI-TOF was correct in 98.4% (65/66) of NTM isolated in our clinical practice (*M. avium, M. intracellulare, M. abscessus, M. chelonae, M. fortuitum, M. mucogenicum, M. kansasii, and M. scrofulaceum*). MALDI-TOF was found to be an accurate, rapid, and cost-effective system for identification of mycobacteria species.

## 1. Introduction

Nontuberculous mycobacteria (NTM) are environmental organisms found in soil and water throughout the world. The large majority of NTM are not pathogenic for humans, but almost all can behave as opportunists and thus be responsible for disease in the presence of predisposing conditions. Presentation is typically pulmonary, skin/soft tissue, lymphatic, or disseminated [1, 2]. The incidence of diseases caused by this group of pathogens is on the rise, due among other reasons to an increase in immunocompromised patients [3, 4]. The distribution of NTM in our environment and the high variability in the pathogenicity of some species means that it is important to differentiate them clinically depending on whether they arise in an asymptomatic setting, due to environmental contamination, or are real infections, in order to apply appropriate antimicrobial therapies. Rapid and accurate diagnosis of mycobacterial infection is of utmost importance as inappropriate treatment may lead to drug resistance or unnecessary exposure to drug toxicity. The American Thoracic Society and Infectious Disease Society of America (ATS/IDSA) recommended that clinically significant NTM isolates be identified to the species level whenever possible [5].

Historically, species-level identification of NTM was a long and complicated process. Growth characteristics in culture (development of color and grow rate) and substrate utilization were for decades the only methods available and sometimes no accurate identification was possible. High-performance liquid chromatography (HPLC) analysis of mycolic acid has been used, but this method is labor-intensive and requires initial culture of isolates on solid medium [6–8]. In the last decades new strategies have been developed using molecular techniques. Currently, nucleic acid sequencing is the most rapid and accurate method for identifying* Mycobacterium* species; however, species-level discrimination may require analysis of several genes [6, 9] and, as with HPLC, requires a pure isolate obtained from solid medium, which delays the turnaround time. Thus, these methods remain limited to specialized laboratories. Tests based on hybridization directly from liquid culture medium are available in many laboratories, although the number of species that can be identified is limited. GenoType* Mycobacterium* CM/AS allows the detection of 36 species of NTM. It is a technology that can be performed in solid and liquid cultures, using the same PCR product [10, 11].

Matrix-assisted laser desorption ionization-time of flight mass spectrometry (MALDI-TOF MS) is recognized as a powerful tool for the identification of bacteria and yeasts in the clinical laboratory [12, 13]. This technique allows identification of organisms on the basis of unique spectral fingerprints produced by extracted proteins. The method is relatively simple, rapid, and associated with significantly lower consumable costs than traditional microbiological identification methods. Although the MALDI-TOF MicroFlex LT mass spectrometer and associated software are expensive initially (approximately $200,000), the continuing consumable costs are inexpensive (less than $1 per isolate). Some authors have used MALDI-TOF for rapid identification of* Mycobacterium* species [6, 14–16], but few have undertaken prospective studies using clinical isolates [16].

We undertook a prospective study with NTM recovered from clinical samples in the routine practice of a microbiological laboratory and demonstrated the usefulness of MALDI-TOF for the daily identification of mycobacteria in our laboratory. We here compare the results of MALDI-TOF and GenoType* Mycobacterium* CM/AS for the identification of NTM from clinical specimens.

## 2. Materials and Methods

Between July 2013 and July 2014 we studied NTM isolates in the microbiology laboratories of the Regional University Hospital and the Virgen de la Victoria University Hospital, both in Malaga, Spain.

### 2.1. *Mycobacterium* Isolates

A total of 66 isolates (from 66 patients) from different types of clinical specimens (mainly respiratory) were analyzed. Samples from nonsterile sites were treated with N-acetyl cysteine and NaOH and cultured on Lowenstein-Jensen medium (LJ) and in a mycobacterium growth indicator tube (MGIT) (Becton Dickinson Microbiology Systems, Cockeysville, MD) and incubated in Bactec MGIT 960. Blood and bone marrow were inoculated into a vial of Myco/F Lytic (Becton-Dickinson, Sparks, MD). In positive cultures, Ziehl-Neelsen staining was performed to confirm the presence of acid-fast bacilli. GenoType was performed using liquid culture medium MGIT and identification using MALDI-TOF was made from colonies isolated on Lowenstein solid culture medium.

In the case of nonidentification of mycobacteria by either of the methods, the strains were sent to the* Mycobacterium* Reference Laboratory at the National Center of Microbiology (CNM) in Majadahonda (Madrid), where identification of the strains was performed by genotypic methods.

### 2.2. Mycobacteria Extraction Protocol for MALDI-TOF MS

We used a modification of the Inactivated Mycobacteria bead preparation method (inMBpm) of Bruker Daltonics [17], described below.

We use a fresh culture from a Lowenstein medium with enough biomass to undertake the process. In a biological safety cabinet we transfer enough colonies to obtain a 5 *μ*L pellet and, with the help of a sterile swab, transfer it to an Eppendorf tube with 300 *μ*L of water (HPLC grade). This is then inactivated for 30 minutes at 95°C in a thermoblock. At this point, we centrifuge at the maximum speed (13.000–15.000 rpm) for 2 minutes, after which the supernatant is removed. We add 300 *μ*L of water (HPLC grade) and mix the sediment carefully. We then add 900 *μ*L of 100% ethanol and mix it again using vortex. We centrifuge it once more at the maximum speed (13,000–15,000 rpm) for 2 minutes after which we remove the supernatant. Afterwards, we resuspend the pellet in 500 *μ*L of water (HPLC grade), centrifuge it at maximum speed (13,000–15,000 rpm) for 2 minutes, and remove the supernatant. We resuspend the pellet in 50 *μ*L of water (HPLC grade) and heat it for 10 minutes at 95°C in a thermoblock. When the sample has cooled, we add 1200 *μ*L of absolute alcohol previously stored in a freezer (−18°C/−20°C) and the mixture is centrifuged at the maximum speed (13,000–15,000 rpm) for 2 minutes. Subsequently we remove all the supernatant and leave the pellet to dry for 5 minutes with the tube open to remove the ethanol completely.

Using the tip of a small spatula we add silica beads (0.5 mm zirconia/silica beads) and 20 *μ*L of pure acetonitrile and mix well for one minute in vortex. We then add 20 *μ*L of 70% formic acid and centrifuge this at the maximum speed (13,000–15,000 rpm) for 2 minutes. From each sample, 1 *μ*L of supernatant is placed in three of the 96 spots of the steel target plate (Bruker) and is allowed this to dry at room temperature. Finally, we add 1 *μ*L of HCCA matrix solution (*α*-cyano-4-hydroxycinnamic acid) and leave this to dry before further analysis by MALDI-TOF MS. Each sample is analyzed in triplicate, using the highest score for further analysis.

### 2.3. MALDI-TOF MS Analysis

Spectra are acquired in a linear positive ion mode at a laser frequency of 60 Hz across a mass/charge ratio (*m/z*) of 2,000 to 20,000 Da using the Microflex LT MALDI-TOF MS (Bruker Daltonik GmbH, Bremen, Germany). The protein profile is obtained by the software FlexControl 3.3 (Bruker Daltonik GmbH, Bremen, Germany) and analyzed by the program FlexAnalysis 3.3 (Bruker Daltonik GmbH, Bremen, Germany). The Mycobacteria Library v 1.0 is used, containing 173 mycobacterial protein profiles, representing 94 species. We consider a range of between 2.0 and 3.0 as acceptable, and scores between 1.6 and 2.0 are considered consistent when the same identification is repeated in most of the 10 possibilities provided by the project. Lower scores (1.7-1.6) are reported to give a correct identification [18–20].

### 2.4. GenoType* Mycobacterium* CM/AS

GenoType* Mycobacterium* CM/AS is a commercial DNA strip assay used for the identification of mycobacteria. These two strips, CM (“Common Mycobacteria”) and GenoType AS (“Additional Species”), are designed to identify different patterns, of which 23 patterns can be assigned to single species and 8 patterns are allocated to two or more* Mycobacterium* species.

### 2.5. Molecular Identification in* Mycobacterium* Reference Laboratory

Strains sent to the* Mycobacterium* Reference Laboratory were identified by typing by polymerase chain reaction (PCR) followed by restriction fragment length polymorphism (RFLP) analysis with BstEII and HaeIII restriction enzymes of gene hsp-65 [21], using the updated band patterns that can be found on the web page (http://app.chuv.ch/prasite/index.html), and sequencing and analysis of gene 16S rRNA.

### 2.6. Statistical Analysis

To study the degree of concordance between the two methods we used the Spearman correlation and the Kappa coefficient. A *P* < 0.05 was considered statistically significant. The analyses were done with PSPP (0.8.3) for Mac.

We have considered the identifications of species closely related inside some complexes to be concordant:* M. phocaicum-mucogenicum, M. porcinum-fortuitum, M. intracellulare-chimaera*, and* M. parascrofulaceum-scrofulaceum*.

## 3. Results

We obtained result with MALDI-TOF in 65 and with Genotype in 64 of the 66 MNT which were studied.

MALDI-TOF MS generated acceptable confidence scores (score > 2.000 or >1.600 if the same species was repeated in the 10 possibilities given by the project) for 65 (98.5%) isolates: 16* M. avium,* 13* M. intracellulare,* 6* M. fortuitum,* 5* M. chelonae,* 4* M. abscessus*, 3* M. phocaicum*, 4* M. kansasii,* 4* M. gordonae,* 3* M. lentiflavum,* 2* M. parascrofulaceum,* 1* M. peregrinum,* 1* M. porcinum,* 1* M. marinum,* 1* M. gastri*, and 1* M. elephantis*. This included 49 (74.2%) identified with a score > 2.000 and 17 (25.7%) with a score between 1.600 and 2.000.

Scores obtained for the more frequently isolated species were* M. avium* (75% > 2.000, 87.5% > 1.800),* M. intracellulare* (84.6% > 2.000),* M. fortuitum* (85.7% > 2.000, 100% > 1.800),* M. chelonae* (40% > 2.000, 60% > 1.800),* M. abscessus* (100% > 2.000), and* M. kansasii* (50% > 2.000, 75% > 1.800).

**Figure 1:**
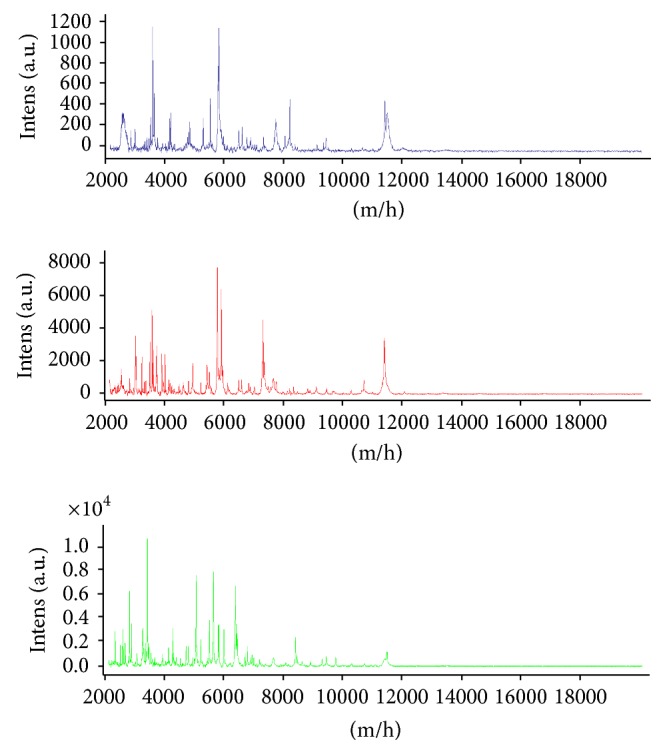
Protein profile of the most common species isolated (*M. intracellulare, M. avium, *and* M. fortuitum*).

Protein profile of the most common species isolated is represented in Figure 1.

The results obtained by GenoType were 16* M. avium*, 13* M. intracellulare*, 7* M. fortuitum*, 5* M. chelonae*, 4* M. abscessus*, 3* M. phocaicum*, 4* M. kansasii*, 4* M. gordonae* 3* M. lentiflavum*, 2* M. scrofulaceum*, 1* M. peregrinum*, 1* M. marinum*, 1* M. gastri*, and 1* Mycobacterium* genus; in one no* Mycobacterium* genus was found. This strain was identified as* M. elephantis* by MALDI-TOF (score 1.744) and also by sequencing at the* Mycobacterium* Reference Laboratory. One strain identified as* Mycobacterium* genus by GenoType was not recognized by MALDI-TOF and was identified as* M. duvalii* by the sequencing method at the* Mycobacterium* Reference Laboratory.

The strains identified using MALDI-TOF as* M. phocaicum* were identified as* M. mucogenicum* by GenoType. A strain identified with MALDI-TOF as* M. porcinum* was identified as* M. fortuitum* by GenoType. Two strains were identified by MALDI-TOF as* M*.* intracellulare/M. chimaera* (both identifications alternating in all the results [10] of each project) and recognized by GenoType as* M. intracellulare*. Two strains were identified by MALDI-TOF as* M*.* scrofulaceum/parascrofulaceum* (both identifications alternating in all the results [10] of each project) and identified by GenoType as* M. scrofulaceum*. The results are shown in Table 1.

The concordance analysis between the two methods (MALDI-TOF and GenoType) showed agreement in 64 of the 66 cases (96.9%). In one case no identification was made with either of the two methods and the other was identified as* M. elephantis* by MALDI-TOF but was not identified by GenoType. The Spearman correlation was 0.997 (*P* < 0.001) and the Kappa value was 0.965 (*P* < 0.001).

## 4. Discussion

The usefulness of MALDI-TOF for the identification of common bacteria has already been demonstrated [12, 13, 22, 23]. However, its main aim is to improve the identification of fungi (yeast and filamentous fungi) and mycobacteria, which have traditionally been the most difficult microorganisms to identify.

Molecular methods for the study of mycobacteria have resulted in great improvement in species identification and have permitted the description of new species. However, these techniques are not available in all laboratories because of their cost and complexity. Whilst commercial hybridization methods are available in most laboratories, their capacity to identify different NTM species is limited (fewer than 40 species), though there are more than 150 recognized species of NTM. Infections produced by mycobacteria are still a great problem, particularly tuberculosis, as are those caused by NTM, which mainly affect children, immunosuppressed persons, and patients with other pathologies like cystic fibrosis. The MALDI-TOF can provide laboratories with a new technique for the identification of mycobacteria, especially given its simplicity and the large number of species included in its database.

Although the number of strains in our study was not large (66) and the number of species was small (15), our results show that identification using MALDI-TOF is correct in the majority of clinically relevant strains of NTM (*M. avium, M. intracellulare, M. abscessus, M. chelonae, M. fortuitum, M. mucogenicum, M. kansasii, *and* M. scrofulaceum*), showing a good agreement with GenoType (96, 9%).

In the three isolates that were identified by GenoType as* M. mucogenicum*, the result obtained by MALDI-TOF was* M. phocaicum*, which cannot be identified by GenoType.* M. phocaicum* belongs to the* Mycobacterium mucogenicum *group (*M. mucogenicum*,* M. phocaicum*,* M. aubagnense*, and* M. llatzerense*) [14, 24, 25], which has such genetic similitude as to make it difficult to distinguish one from the other, even using sequencing techniques [26]. One of the strains identified by GenoType as* M. fortuitum* was identified by MALDI-TOF as* M. porcinum*, a species that GenoType is unable to identify. This species belongs to the group known as* Mycobacterium fortuitum* (*M. fortuitum, M. peregrinum, M. senegalese, M. mageritense, M. septicum, M. alvei, M. houstonense, M. boenickei, M. conceptionense, M. porcinum, M. neworleansense*, and* M. brisbanense*) [25]. Some studies have shown almost complete phenotypic and molecular identity between clinical isolates of the* M. fortuitum* group and strains of* M. porcinum* [27]. Two strains were identified by MALDI-TOF as* M*.* intracellulare/M. chimaera *(both identifications alternating in all the results [10] of each project), being identified by GenoType as* M. intracellulare*. This finding has been described previously because they are two closely related organisms, differing by only 1 base pair in their 16S RNA gene regions [28, 29]. Thus, the ambiguity encountered when these organisms are identified by MALDI-TOF is not surprising. Moreover, it is not clear whether differences exist in the pathogenicity of these two species [18]. Two strains were identified by MALDI-TOF as* M*.* scrofulaceum/parascrofulaceum *(both identifications alternating in all the results [10] of each project), being identified by GenoType as* M. scrofulaceum. *Bruker's mycobacteria Library v 1.0 shows difficulties in the distinction between* M. scrofulaceum *and* M. parascrofulaceum*, likely due to the similarity in the protean profile. These mycobacteria show a similar antibiotic sensitivity profile, so differentiation could be irrelevant in a clinical context [14, 24]. In one strain identified as* M. elephantis* by MALDI-TOF the score was low (1.744), but the species identification was repeated in all the trials and was confirmed in the Reference Laboratory by sequencing. The only strain not identified by MALDI-TOF was identified in the Reference Laboratory as* M. duvalii, *probably because this species is not included in the mycobacteria Library v 1.0.

In most cases identification with MALDI-TOF generated a good score (74.5% > 2.000), especially in species with greater clinic relevance:* M. avium* (75% > 2.000, 87.5% > 1.800),* M. intracellulare* (84.6% > 2.000),* M. abscessus* (100% > 2.000), and* M. kansasii* (50% > 2.000, 75% > 1.800). In our opinion, it is important to evaluate both the score and the repetition of the result.

Previous studies have demonstrated the potential of MALDI-TOF for accurate* Mycobacterium *identification, both* M. tuberculosis* complex and NTM [6, 8, 15, 16, 18, 30]. Some authors found differences in the correct identification percentage according to the culture media from which the strains were obtained [6, 8, 30], the culture growth time [14], the extraction protocol applied to the mycobacteria [6, 15, 30], or the library used [6]. Our results were obtained from isolates on Lowenstein media, most of them recent cultures, though late growth cultures were used in some cases, which could explain the worse quality spectra.

There is no doubt that the appropriate extraction protocols will be optimized, especially in liquid culture media, in order to reduce the time to diagnosis. There is now a new Mycobacteria Library (v 2.0), but it is still necessary to optimize and update it with new species, and it should be able to discriminate the different species of the* M. Tuberculosis Complex*, which is not possible at present.

To date, NTM species level identification has been limited to specialized laboratories. The MALDI-TOF offers quick results, is easy to perform, and involves a low cost for reagents. Also, the procedure involves little handling and few working hours, which reduces the potential risk of acquiring infections caused by mycobacteria in the laboratory.

Our study supports the idea that MALDI-TOF is a suitable, reliable, and fast technique for identification of NTM. However, it is important to standardize the procedure in liquid media inoculated with clinical specimens to reduce the time to diagnosis. Accordingly, further study is required to validate these results in clinical practice.

## Figures and Tables

**Table 1:** Table of results.

	GenoType	MALDI-TOF	Score		GenoType	MALDI-TOF	Score	Sequencing
1	*M. avium *	*M. avium *	2.078	34	*M. fortuitum *	*M. fortuitum *	2.157	
2	*M. avium *	*M. avium *	1.657	35	*M. fortuitum *	*M. fortuitum *	1.877	
3	*M. avium *	*M. avium *	1.932	36	*M. fortuitum *	*M. porcinum *	2.222	
4	*M. avium *	*M. avium *	2.041	37	*M. chelonae *	*M. chelonae *	2.307	
5	*M. avium *	*M. avium *	1.626	38	*M. chelonae *	*M. chelonae *	1.667	
6	*M. avium *	*M. avium *	2.101	39	*M. chelonae *	*M. chelonae *	1.857	
7	*M. avium *	*M. avium *	2.100	40	*M. chelonae *	*M. chelonae *	2.064	
8	*M. avium *	*M. avium *	2.038	41	*M. chelonae *	*M. chelonae *	1.715	
9	*M. avium *	*M. avium *	2.009	42	*M. abscessus *	*M. abscessus *	2.107	
10	*M. avium *	*M. avium *	2.038	43	*M. abscessus *	*M. abscessus *	2.060	
11	*M. avium *	*M. avium *	1.858	44	*M. abscessus *	*M. abscessus *	2.320	
12	*M. avium *	*M. avium *	2.191	45	*M. abscessus *	*M. abscessus *	2.198	
13	*M. avium *	*M. avium *	2.100	46	*M. mucogenicum *	*M. phocaicum*	2.264	
14	*M. avium *	*M. avium *	2.180	47	*M. mucogenicum *	*M. phocaicum*	2.027	
15	*M. avium *	*M. avium *	2.003	48	*M. mucogenicum *	*M. phocaicum*	2.150	
16	*M. avium *	*M. avium *	2.009	49	*M. kansasii *	*M. kansasii *	1.600	
17	*M. intracellulare *	*M. intracellulare *	2.168	50	*M. kansasii *	*M. kansasii *	1.891	
18	*M. intracellulare *	*M. intracellulare *	2.217	51	*M. kansasii *	*M. kansasii *	2.199	
19	*M. intracellulare *	*M. intracellulare *	1.600	52	*M. kansasii *	*M. kansasii *	2.257	
20	*M. intracellulare *	*M. intracellulare *	2.005	53	*M. gordonae *	*M. gordonae *	1.914	
21	*M. intracellulare *	*M. intracellulare *	2.182	54	*M. gordonae *	*M. gordonae *	2.232	
22	*M. intracellulare *	*M. intracellulare *	2.148	55	*M. gordonae *	*M. gordonae *	2.096	
23	*M. intracellulare *	*M. intracellulare *	2.275	56	*M. gordonae *	*M. gordonae *	2.274	
24	*M. intracellulare *	*M. intracellulare *	2.272	57	*M. lentiflavum *	*M. lentiflavum *	2.171	
25	*M. intracellulare *	*M. intracellulare/Ch^1^*	2.016	58	*M. lentiflavum *	*M. lentiflavum *	2.205	
26	*M. intracellulare *	*M. intracellulare *	2.369	59	*M. lentiflavum *	*M. lentiflavum *	2.278	
27	*M. intracellulare *	*M. intracellulare *	2.057	60	*M*. *scrofulaceum *	*M. scrofulaceum/para^2^*	**1.985**	
28	*M. intracellulare *	*M. intracellulare/Ch^1^*	1.730	61	*M*. *scrofulaceum *	*M. scrofulaceum/para^2^*	**1.900**	
29	*M. intracellulare *	*M. intracellulare *	2.050	62	*M. peregrinum *	*M. peregrinum *	2.056	
30	*M. fortuitum *	*M. fortuitum *	2.376	63	*M. marinum *	*M. marinum *	2.046	
31	*M. fortuitum *	*M. fortuitum *	2.169	64	*M. gastri *	*M. gastri *	**2.270**	
32	*M. fortuitum *	*M. fortuitum *	2.643	65	No mycobacteria	*M. elephantis *	1.744	*elephantis *
33	*M. fortuitum *	*M. fortuitum *	2.223	66	*Mycobacterium *sp.	Unidentified		*duvalii *

^1^ *M. intracellulare/M. chimaera*.

*^2^M. scrofulaceum/parascrofulaceum*.
